# Faricimab efficacy in type 1 macular neovascularization: AI-assisted quantification of pigment epithelium detachment (PED) volume reduction over 12 months in Naïve and switch eyes

**DOI:** 10.1186/s40942-025-00629-w

**Published:** 2025-01-09

**Authors:** Jennifer Cattaneo, Paolo Forte, Giovanni Forte, Chiara M. Eandi

**Affiliations:** 1 Fondation Asile des Aveugles, Department of Ophthalmology, Jules-Gonin Eye Hospital, University of Lausanne, Avenue de France 54, Lausanne, 1001 Switzerland; 2 Eye Unit, IRCCS Ospedale Policlinico San Martino, Genoa, Italy; 3 DINOGMI, University of Genoa, Genoa, Italy; 4 School of Medicine, University of Pavia, Pavia, Italy; 5 Department of Surgical Sciences, University of Torino, Torino, Italy

**Keywords:** Type 1 macular neovascularization, Type 1 MNV, Pigment epithelium detachment, PED, Faricimab, Intravitreal injection, Artificial intelligence (AI), OCT, Retina, Choroid, Imaging

## Abstract

**Background:**

This study evaluates the efficacy of intravitreal Faricimab in reducing pigment epithelium detachment (PED) and fluid volumes in both treatment-naïve eyes and eyes unresponsive to anti-VEGF mono-therapies, all diagnosed with type 1 macular neovascularization (T1 MNV) over a period of 12-month.

**Methods:**

A retrospective, single-center cohort study was conducted at the Jules Gonin Eye Hospital, Lausanne, Switzerland. Clinical records of treatment-naïve and non-responder switch patients presenting T1 MNV secondary to neovascular age-related macular degeneration (nAMD) from September 2022 to March 2023 were reviewed. Patients received a loading dose of three monthly Faricimab injections followed by a treat-and-extend (T&E) regimen. Multimodal imaging, including structural OCT and AI-assisted analysis, was used to quantify PED volumes and related fluid biomarkers at baseline, 3-month, 6-month, and 12-month follow-up. Statistical analyses included linear mixed models to evaluate differences and trends in intraretinal (IRF), subretinal fluid (SRF) and PED volumes.

**Results:**

65 eyes of 65 patients were enrolled (female: 70.7%; mean age = 80.7yrs, SD = 6.9yrs). 80% had received anti-VEGF treatment (Switch group) and 20% were treatment-Naïve at baseline. At 12 months, intravitreal treatments were more frequent in the Switch group (mean number = 8.3 vs. 6.0; *p* = 0.009). BCVA improved at the 12-month follow-up in Naïve eyes (+ 6.9 ETDRS letters from baseline, *p* = 0.053) and was maintained in Switch eyes. No cases of intraocular inflammation were observed. Significant reduction in SRF and IRF volumes were noted in both groups. A significant reduction in PED volume was observed over the follow-up period in both groups (mean slope = -206 nL, 95%CL = -273/-138; *p*-value < 0.001).

**Conclusions:**

Intravitreal Faricimab significantly reduced PED volumes in both treatment-Naïve and non-responder Switch patients over 12 months. The study highlights Faricimab’s potential as an effective treatment option for T1 MNV in nAMD, offering significant improvements in PED volume and related fluid biomarkers.

## Background

Type 1 macular neovascularization (T1 MNV) is the most frequent MNV subtype occurring in neovascular age-related macular degeneration (nAMD) [1] and it is defined as an ingrowth of neovessels into and within the sub-RPE space from the choriocapillaris (CC) [2]. The feeding and drainage vessels inside both the choroid and the lesion undergo changes and enlargement as the lesion grows and expands; this mechanism consequently leads to clinical exudation, manifesting as subretinal fluid (SRF), intraretinal fluid (IRF), and subretinal hyper-reflective material (SHRM) [3]. Another feature of T1 MNV is the occurrence of retinal pigment epithelium detachments (PEDs), which are defined as a clinically visible separation of the retinal pigment epithelium (RPE) monolayer from the underlying Bruch’s membrane (BM) [4]. The presence of PEDs complicates the prognosis of patients with nAMD due to the risk of RPE tear [5], and the development of macular atrophy [6] and macular fibrosis [7, 8].

The standard of treatment for T1 MNV complicated by the presence of PEDs is intravitreal injections of anti-vascular endothelial growth factor (anti-VEGF) agents [9]. Nevertheless, despite the wide range of available molecules, a considerable proportion of patients demonstrates resistance to treatment or a partial response, necessitating additional or alternative approaches to treatment [10]. 

In 2022, the first humanized, bispecific IgG monoclonal antibody, Faricimab (Vabysmo, RG7716, Roche/Genentech), which inhibits both VEGF-A and Ang-2, was approved for nAMD after two phase III clinical trials (TENAYA and LUCERNE) [11]. The anti-VEGF-A effect inhibits endothelial proliferation, reduces vascular permeability, and suppresses neovascularization. The anti-Ang 2 effect improves vascular stability and desensitizes the vessels to the actions of VEGF-A [12]. Phase III studies have demonstrated that Faricimab administered up to every 16 weeks was non-inferior to Aflibercept administered every 8 weeks, with a comparable reduction in central retinal thickness and improvement in best-corrected visual acuity (BCVA) in treatment-naïve patients [13]. In a post hoc analysis of pooled data from the TENAYA and LUCERNE trials, Faricimab was associated with greater reduction in PEDs during the head-to-head dosing period of the pivotal trials (Avery et al., presented at Macula Society Annual meeting, 2024). Few studies have investigated the clinical and anatomical outcomes of patients with suboptimal response to anti-VEGF mono-therapies, who were switched to Faricimab [14], in particular evaluating the short-term changes in maximum PED height [15, 16].

Recent advances in the use of artificial intelligence (AI) for automated quantitative analysis of retinal images have provided an innovative way to investigate clinical and topographical characteristics of nAMD, enabling the discovery of potential novel features for disease prognosis, detection, monitoring, and prognosis [17]. ^,^ [18]

This study aims to explore the efficacy of intravitreal Faricimab in reducing PED volumes, quantified by an AI-assisted algorithm, over a 12-month period in both treatment-naïve and non-responder switch eyes. In order to better characterize and interpret the volume-based predictive outcomes, we specifically focused our analysis on T1 MNV, in which PED volume may serve as an indicator of fluid accumulation arising from active MNV components within the materials in PEDs.

## Methods

### Study design and population

In this retrospective, single-center, cohort study we reviewed the clinical records of treatment-naïve and non-responder switch patients diagnosed with T1 MNV secondary to exudative nAMD at baseline, 3-month, 6-month and 12-month follow-up after intravitreal Faricimab injections. The electronic medical record system of the Medical Retina Department of the Jules Gonin Eye Hospital, Lausanne, Switzerland, was searched using codes for faricimab between September 2022 and March 2023 and cross-referenced with the list of patients diagnosed with Type 1 MNV complicated by PED. Therefore, patients with only Type 1 MNV, without PED, were not included in the database search. The study protocol and analysis adhered to the tenets of the Declaration of Helsinki and the research protocol was approved by the local Ethical Committees (CERVD: 2017 − 00493). Enrolled patients signed informed consent.

The eligibility criteria included: (1) patients aged > 55 years; (2) diagnosis of T1 MNV with PED at baseline secondary to nAMD. In accordance with previous studies, PED was defined as a pigment epithelial elevation greater than 400 μm in width and 75 μm in height, or over 200 μm in height on spectral-domain optical coherence tomography (SD-OCT) [19]; (3) a minimum of 12-month follow-up; and (4) both naïve and switch patients treated with a loading dose of 3 monthly Faricimab injections (6 mg/0.05 ml) followed by a treat and extend (T&E) regimen. In case of any evidence of exudation, such as the presence of SRF, IRF and SHRM as detected by structural OCT, or a newly detected hemorrhage on fundus examination, the interval was reduced by 4-week increments. In absence of signs of active exudation, treatment intervals were extended by 4 weeks. Non-responder patients were switched to intravitreal (IVT) Faricimab in case of persistence of fluid after at least 6 previous anti-VEGF injections (Ranibizumab or Aflibercept 2 mg) or recurrence at 8 weeks interval. The following conditions were excluded from the study: (1) history of any other chorioretinal disease (such as history of retinal detachment, vitrectomy, retinal vascular diseases) or optic nerve disorders (such as advanced glaucoma, or optic nerve disease resulting in significant optic nerve alterations limiting visual function); (2) relevant optic media opacities and/or insufficient fixation to allow high-quality imaging; and (3) myopia > 6 diopters (D) of sphere or 3 D of cylinder and/or axial length > 25.5 mm; (4) recent history of ocular procedures (less than 90 days since cataract extraction with intraocular lens implantation or less than 30 days since YAG laser capsulotomy).

### Data collection

Multimodal imaging studies were reviewed by two independent and masked readers (J.C. and P.F.). In addition to the demographic features and laterality, the following clinical findings were recorded: best-correct visual acuity (BCVA) using ETDRS letters assessed using a retro-illuminated (160 cd/m^2^) ETDRS charts at 4 m (number of letters), fellow-eye status (iAMD, nAMD or cRORA), drusen phenotype (soft drusen, subretinal drusenoid deposits or both), number of Faricimab injections during the follow-up period. All patients underwent a multimodal imaging evaluation that included SLO-infrared images (IR), structural OCT (Spectralis HRA + OCT; Heidelberg Engineering, Heidelberg, Germany), and as a standard of care in our clinical practice at baseline was performed at least one ancillary imaging modality between OCT-angiography (Spectralis HRA + OCT), fluorescein angiography (FA) and indocyanine green angiography (ICGA) (Spectralis HRA + OCT) to minimize the risk of misdiagnosis. OCT scans were obtained with a volumetric acquisition (20°x20° field, 97 sections, Automatic Real Time Function 12 scans).

### OCT grading

**Fig. 1:**
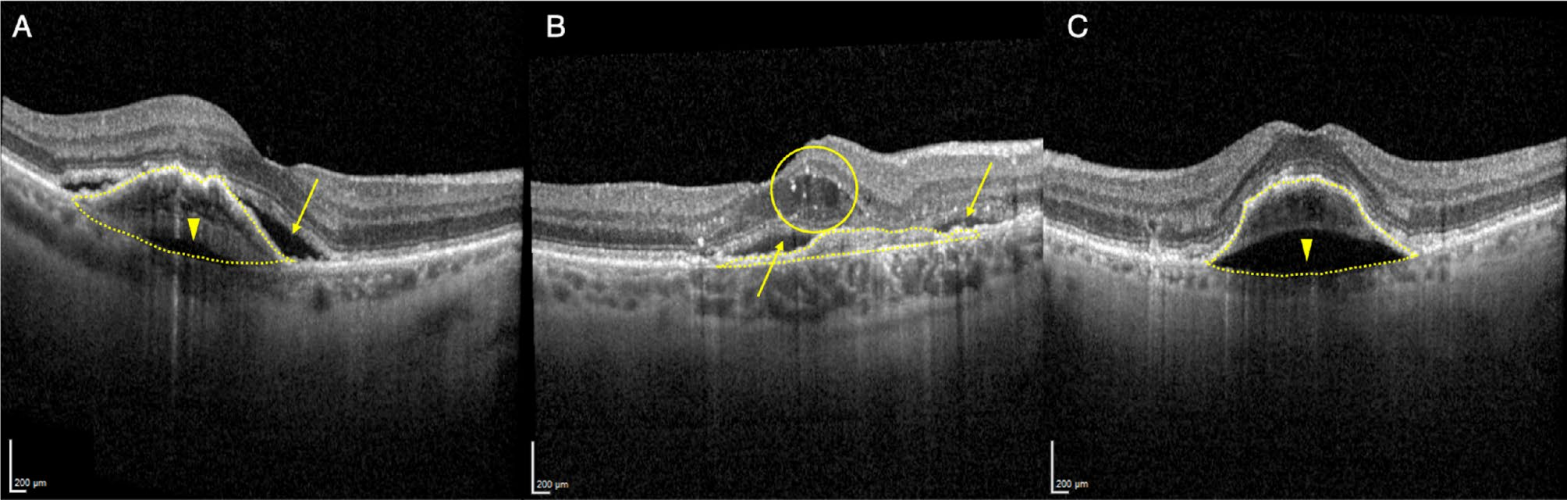
Representation of the optical coherence tomography (OCT) biomarkers assessed in the study. (**A**) Type 1 Macular Neovascularization (T1 MNV) is characterized by an ingrowth of neovessels in the sub-RPE space leading to varying types of pigment epithelium detachments (PEDs): dashed line delineates a fibrovascular PED associated with outward exudation (arrowhead) and typical subretinal fluid (SRF; arrow) at the edge of the neovascular complex (dashed lines); (**B**) accumulation of fluid may also occur in the intraretinal space (inward exudation) with the creation of intraretinal fluid (IRF) and cystoid spaces (circle), subretinal fluid (SRF) is indicated with arrows; (**C**) T1 MNV may also present with a predominantly outward exudation, even in absence of a posterior bowing at theBruch’s Membrane (arrowhead). In particular, PED volume (dashed lines) was considered as an indicator of the fluid accumulation arising from active MNV components i.e., intralesional + outward fluid component

The following OCT biomarkers were graded at each follow-up visit by two blinded readers (JC and PF): (1) T1 MNV features of inward exudation with reference to the RPE layer, specifically intraretinal fluid (IRF), subretinal fluid (SRF), and subretinal hyperreflective material (SHRM) [2]; (2) T1 MNV features of outward exudation with reference to the RPE layer: the presence of a lenticular hyporeflective space located between Bruch’s Membrane (BM) and the base of the fibrovascular MNV complex, also reported as prechoroidal cleft [18, 19] (Fig. 1). We considered this biomarker as a sign of inadequate control of sub-RPE exudation; (3) maximum PED Height, measured manually as the greatest vertical distance between RPE layer and BM. The mean value was used for statistical analysis of metric features. For categorical features, in case of disagreement on a single result, a further assessment was performed by a third experienced retina specialist (C.M.E.). PEDs were further classified based on their reflectivity patterns on SD-OCT as follows: predominantly serous PEDs, which contained ≥ 50% serous fluid by volume and displayed optically empty spaces with minimal internal reflectivity; and predominantly fibrovascular PEDs, which consisted of ≥ 50% fibrovascular tissue by volume. After anonymization and blinded to visual results, OCT volumes were analyzed by the AI-driven Discovery^®^ platform (Discovery OCT Fluid and Biomarker Detector, RetinAI AG, Switzerland). This software enables automated segmentation of retinal and choroidal thicknesses, as well as retinal volumes and fluid compartments. The segmented retinal and choroidal layers included: the retinal nerve fiber layer (RNFL), the ganglion cell layer combined with the inner plexiform layer (GCL + IPL), the inner nuclear layer combined with the outer plexiform layer (INL + OPL), the outer nuclear layer (ONL), the photoreceptor layer along with the retinal pigment epithelium (PR + RPE), the choriocapillaris with the choroidal stroma (CC + CS), and the total retinal thickness (CRT, µm). Additionally, the software provides quantitative measurements of subretinal fluid (SRF), intraretinal fluid (IRF), and pigment epithelial detachment (PED) volumes. Once the PED is identified, the AI calculates the area of detachment in each OCT slice and integrates these measurements across all slices to determine the total PED volume within the scanned region.

PED volume was considered as an indicator of the fluid accumulation resulting from active MNV components within the material in PEDs, i.e., intralesional + outward fluid component. When an error in automated thickness and/or volumes was present, a manual annotation was performed.

### Statistical analysis

All statistical analyses and correlations were performed using R (Version 4.3.3). The joint distribution of each categorical was explored using the analysis of contingency tables and described in terms of absolute and relative (%) frequency. Chi-squared test was applied to assess biomarker differences between Naïve and Switch eyes. Metric parameters were described using the mean and standard deviation (SD). On this occasion, the Mann-Whitney test was used to compare the distributions between Naïve and Switch eyes. We compared BCVA between the Switch and Naïve groups at baseline and final follow-up to assess whether the differences between the groups had leveled out, and to determine if any statistically significant differences existed between them. Time tendency of the volumetric analysis (PED volume, SRF volume, and IRF volume from baseline to 3, 6, and 12 months) was performed using a linear mixed model (LMM). All statistics derived from LMM modeling were accompanied by the corresponding 95% confidence limits (95%CL) and a two-sided *p*-value ≤ 0.05 was considered as statistically significant.

## Results

### Descriptive analysis

The study cohort comprised 65 caucasian patients (females: 70.7%; age: mean = 80.7yrs, SD = 6.9yrs) with a total of 65 eyes. Among the study population, 52/65 patients (80.0%) previously received intravitreal anti-VEGF mono-therapy for exudative T1 MNV (mean number of previous IVTs before switch = 40.4 SD = 37.7) without successful control of retinal exudation. The remaining 13/65 cases (20.0%) were treatment-Naïve (Naïve) at baseline and underwent first-line therapy with intravitreal Faricimab. At baseline, there were no significant differences in terms of fellow-eye status (*p*-value = 0.064) and study eye drusen phenotype (*p* = 0.371) with a predominance of soft drusen (Table 1). At baseline, of the total 65 eyes analyzed, 45 (69.2%) presented with fibrovascular PED and 20 (30.8%) with serous PED. The distribution of PED types differed between groups, with fibrovascular PED being more prevalent in the Switch group (38 eyes, 73.1%) compared to the Naïve group (7 eyes, 53.8%), although this difference did not reach statistical significance (*p* = 0.147) (Table 1).

The distribution of categorical and metric parameters according to treatment status is shown in Tables 2 and 3, respectively. At baseline (Table 2), 52 eyes displayed SRF (80.0%), 17 IRF (26.1%), 9 SHRM (13.8%), 8 outwards exudation within the fibrovascular PED (15.4%). Manual assessment of PED Height and Axis, and AI-assisted quantification of PED Volume reduction were comparable between the two groups at baseline (*p*-value > 0.05) (Table 3).

### IVT treatments

The frequency of intravitreal treatments was different between Switch (mean = 8.3, SD = 2.5) and Naïve (mean = 6.0, SD = 2.7) eyes throughout the study, necessitating a more frequent treatment in the Switch group (*p*-value = 0.009). Mean BCVA at baseline was 75.5 and 69.2 letters in the Switch and Naïve group, respectively. At the final follow-up, a favorable BCVA was achieved in both groups (mean = 76.0 and 76.1 ETDRS letters) showing a notable improvement of + 6.9 ETDRS letters from baseline (95% CI: -1.7 to 15.5; *p* = 0.053). No episode of intraocular inflammation (IOI) was registered during the examined interval, while two cases of RPE tear (1 in the group of Switch, 1 in the group of Naïve eyes) occurred after the first treatment and consequently developed cRORA .

### Trend analysis

Regarding the distribution of categorical OCT biomarkers, SRF was present in 80% of eyes at baseline, 21.1% at 3-month, 29.2% at 6-month, and 9.2% at 12-month. A significant decrease was observed for SRF volume across the follow-up (mean slope = -136.2 nL, 95%CL = -188.3/-83.7; *p*-value = 0.038). (Table 4)

IRF was present in 26.1% of eyes at baseline, 7.6% at 3-month, 10.7% at 6-month, and 7.6% at 12-month. The treatment had a positive impact on IRF volume (mean slope = -22.2 nL; 95%CL= -34.3/-9.8, *p*-value = < 0.001).

Outward exudation was present in 13.8% of eyes at baseline, 9.2% at 3-month, 6.1% at 6-month and 4.6% et 12-month (Table 2). A complete resolution of both inward and outward retinal exudation was effectively achieved in 52/65 cases (81.5%) (Table 2).

As reported in Table 3, in Switch eyes at baseline mean CRT was 379 ±187 μm, mean PED height was 246±177 μm, and mean PED volume was 844±995 nL, while at 12-month follow up they decreased to 324±171 μm, 195±152 μm, and 689±841nL, respectively. In Naïve eyes at baseline mean CRT was 463±207 μm, mean PED height was 254±99.3 μm, and mean PED volume was 676±680 nL, and at 12-month follow up they decreased to 263±113 μm, 121±80 μm, 265±259 nL, respectively.

**Fig. 2:**
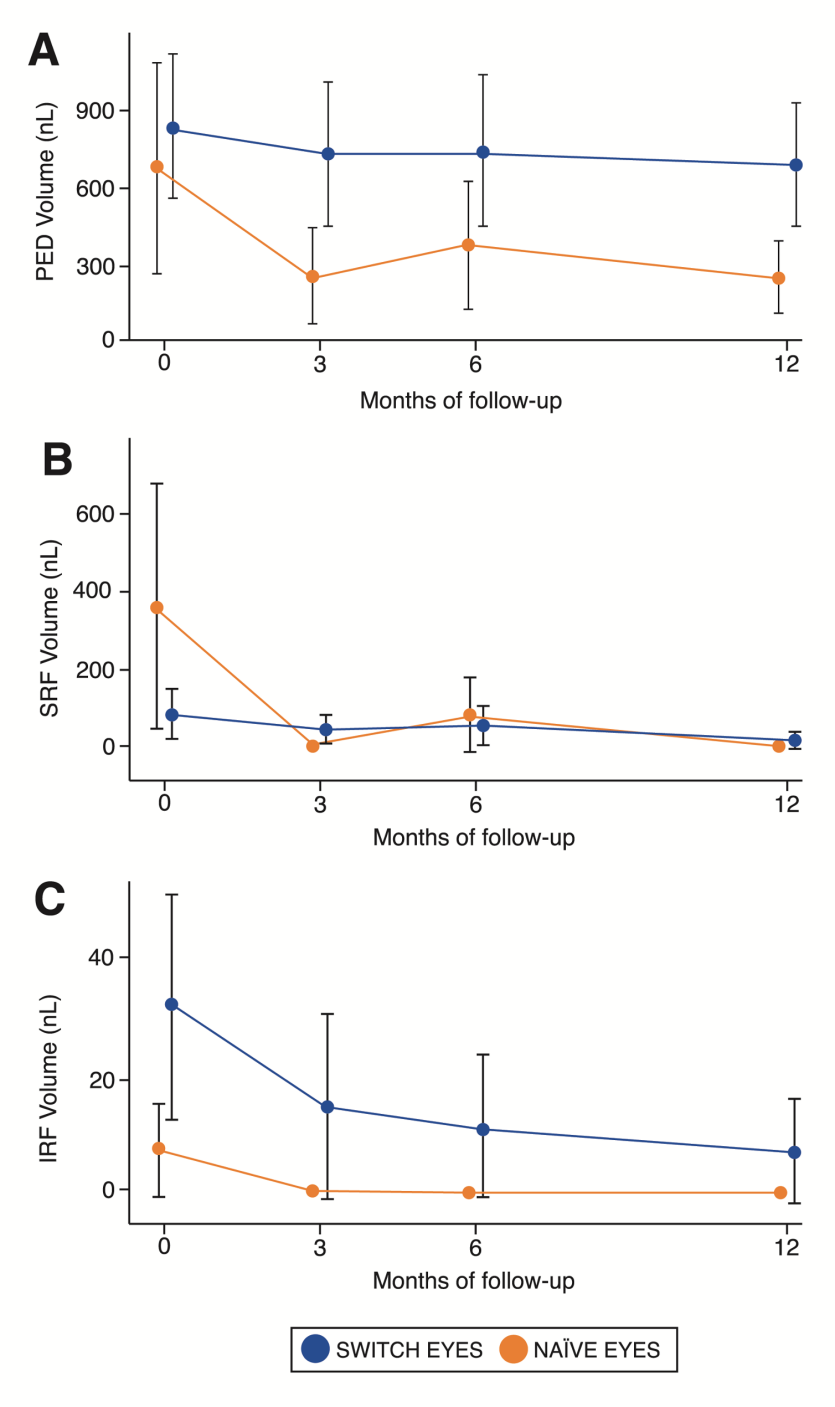
Retinal fluid volumetric changes over 12 months in Switch (blue) and Naïve (orange) eyes treated with Faricimab. (**A**) Pigment epithelium detachment (PED) volume (nL). (**B**) Subretinal fluid (SRF) volume (nL). (**C**) Intraretinal fluid (IRF) volume (nL). Switch eyes exhibit higher baseline PED and IRF volumes, indicative of a chronic disease state. Conversely, Naïve patients show greater SRF volume at baseline. Both groups display a trend of reduction in fluids and PED volume, with a particularly notable early response observed at the 3-month follow-up, sustained at subsequent examinations

A significant reduction in PED volume was observed over the follow-up period (mean slope = -206, 95%CL = -273/-138; *p*-value < 0.001). (Table 4) (Fig. 2).

**Table 1:** Demographics and treatment of the 65 eyes

Categorical Variables		Switch (*N* = 52)		Naïve (*N* = 13)		*P*-value
		*N*	%	*N*	%	
Gender	Male	16	30.7	3	23.1	0.595
	Female	36	69.3	10	76.9	
Fellow-eye Status	iAMD	18	34.6	5	38.5	0.064
	nAMD	25	48.0	8	61.5	
	cRORA	9	17.4	0	0.0	
Drusen Phenotype	Drusen	39	34.6	11	38.5	0.371
	SDD	2	48.0	0	61.5	
	Drusen + SDD	11	17.4	2	0.0	
PED Phenotype	Fibrovascular	38	73.1	7	53.8	0.147
	Serous	14	26.9	6	46.2	
Onset of AEs	RPE tear	1	1.9	1	16.7	0.281 -
	IOI	0	0.0	0	0.0	
Metric Variables		Mean	SD	Mean	SD	*P*-value
BCVA (ETDRS)	Baseline	75.5	9.58	69.2	9.54	0.022*
	12-month	76.0	10.0	76.1	13.7	0.535
IVT (n)	Previous Anti-VEGF	40.4	37.7	0.0	0.00	< 0.001*
	Faricimab	8.3	2.5	6.0	2.7	0.009*

Legenda – iAMD: intermediate age-related macular degeneration; nAMD: neovascular AMD; cRORA: complete RPE and outer retinal atrophy; SDD: subretinal drusenoid deposits.; AEs: adverse events; RPE tear: retinal pigment epithelium tear; IOI: intraocular inflammation; ETDRS: early treatment diabetic retinopathy study; IVT: intravitreal treatment number; Anti-VEGF: anti-vascular endothelial growth factor agents i.e., Aflibercept, Brolucizumab and Ranibizumab; SD: standard deviation; *p*-value: probability level derived by the chi-squared test for categorical variables, and by the Mann-Whitney Test for metric variables;

**Table 2:** Distribution of type 1 macular neovascularization exudative features according to Naïve and switch status

Exudative feature	Follow-up visit	Type 1 MNV		Total (%)	*P*-value
		Switch (*n* = 52)	Naïve (*n* = 13)		
		*N* (%)	*N* (%)		
SRF	Baseline	40 (76.9)	12 (92.3)	52 (80.0)	0.214
	3-month	14 (26.9)	3 (23.1)	17(26.1)	0.777
	6-month	17 (32.7)	2 (15.4)	19 (29.2)	0.219
	12-month	6 (11.5)	0 (0.0)	6 (9.2)	0.198
IRF	Baseline	15 (28.8)	2 (15.3)	17 (26.1)	0.323
	3-month	5 (9.6)	0 (0.0)	5 (7.7)	0.244
	6-month	7 (13.7)	0 (0.0)	7 (10.8)	0.161
	12-month	5 (9.6)	0 (0.0)	5 (7.6)	0.245
SHRM	Baseline	4 (7.6)	5 (38.4)	9 (13.8)	0.004*
	3-month	3 (5.8)	0 (0.0)	3 (4.6)	0.375
	6-month	4 (7.7)	0 (0.0)	4 (6.1)	0.296
	12-month	3 (5.8)	0 (0.0)	3 (4.6)	0.373
Outward Exudation	Baseline	7 (13.4)	1 (7.6)	8 (15.4)	0.571
	3-month	5 (9.6)	1 (7.7)	6 (9.2)	0.832
	6-month	3 (5.8)	1 (7.7)	4 (6.1)	0.795
	12-month	3 (5.8)	0 (0.0)	3 (4.6)	0.373

Legenda – MNV: macular neovascularization; N/%: absolute/relative frequency; SRF: subretinal fluid, SHRM: subretinal hyperreflective material; IRF: intraretinal fluid; *p*-value: probability level derived by the chi-squared test

**Table 3:** Manual and AI-assisted quantification of metric OCT biomarkers and volumetric analysis according to Naïve and switch status

Biomarker	Follow-up	Type 1 MNV		*P*-value
		Switch	Naïve	
		(*N* = 52)	(*N* = 13)	
		Mean (SD)		
CRT (µm)	Baseline	379 (187)	463 (207)	0.118
	3-month	326 (165)	293 (123)	0.920
	6-month	326 (181)	276 (96)	0.755
	12-month	324 (171)	263 (113)	0.234
PED Height (µm)	Baseline	246 (177)	254 (99.3)	0.327
	3-month	210 (155)	130 (100)	0.022*
	6-month	201 (156)	124(74)	0.101
	12-month	195 (152)	121 (80)	0.699
PED Volume (nL)	Baseline	844 (995)	676 (680)	0.727
	3-month	706 (860)	278 (309)	0.049*
	6-month	707 (905)	379 (425)	0.296
	12-month	689 (841)	265 (259)	0.043*

Legenda – OCT: optical coherence tomography; SD: standard deviation; N: absolute frequency; CRT: central retinal thickness; PED: pigment epithelium detachment (Height and Volume); *p*-value: probability level derived by the Mann-Whitney U test

**Table 4:** Time trend of the AI-assisted Volumetric Analysis over the follow-up period through

Factors and categories	Mean	95%CL	Mean difference	95%CL	*P*-value
PED Volume (nL)					< 0.001*
Baseline	810	578/1041	0.0	(Ref.)	
3-month	621	426/815	-189	-256/-122	
6-month	642	438/845	-168	-235/-101	
12-month	604	598/977	-206	-273/-138	
SRF Volume (nL)					0.038*
Baseline	145.7	77.0/214.9	0.0	(Ref.)	
3-month	21.1	5.01/37.1	-124.6	-176.8/-72.3	
6-month	48.2	10.5/85.8	-97.55	-149.8/-45.3	
12-month	9.74	1.35/18.1	-136.02	-188.3/-83.7	
IRF Volume (nL)					< 0.001*
Baseline	27.8	11.4/44.2	0.0	(Ref.)	
3-month	11.9	-0.7/24.7	-15.8	-28.1/-3.5	
6-month	9.9	-0.4/20.2	-17.9	-30.1/-5.6	
12-month	5.7	-01.5/12.9	-22.2	-34.3/-9.8	

Legenda – 95%CL: 95% confidence limits for means. Ref.: reference category; *P*-value: probability level derived from the likelihood ratio test applied to a random-effects linear model

## Discussion

This study investigated the efficacy of intravitreal Faricimab treatment in controlling inward and outward exudation in T1 MNV, particularly focusing on PED volumes. The dual inhibition of VEGF-A and Ang-2 of Faricimab offers advantages over existing anti-VEGF mono-therapies, especially in cases where resistance or inadequate response to conventional treatments is encountered.

**Fig. 3:**
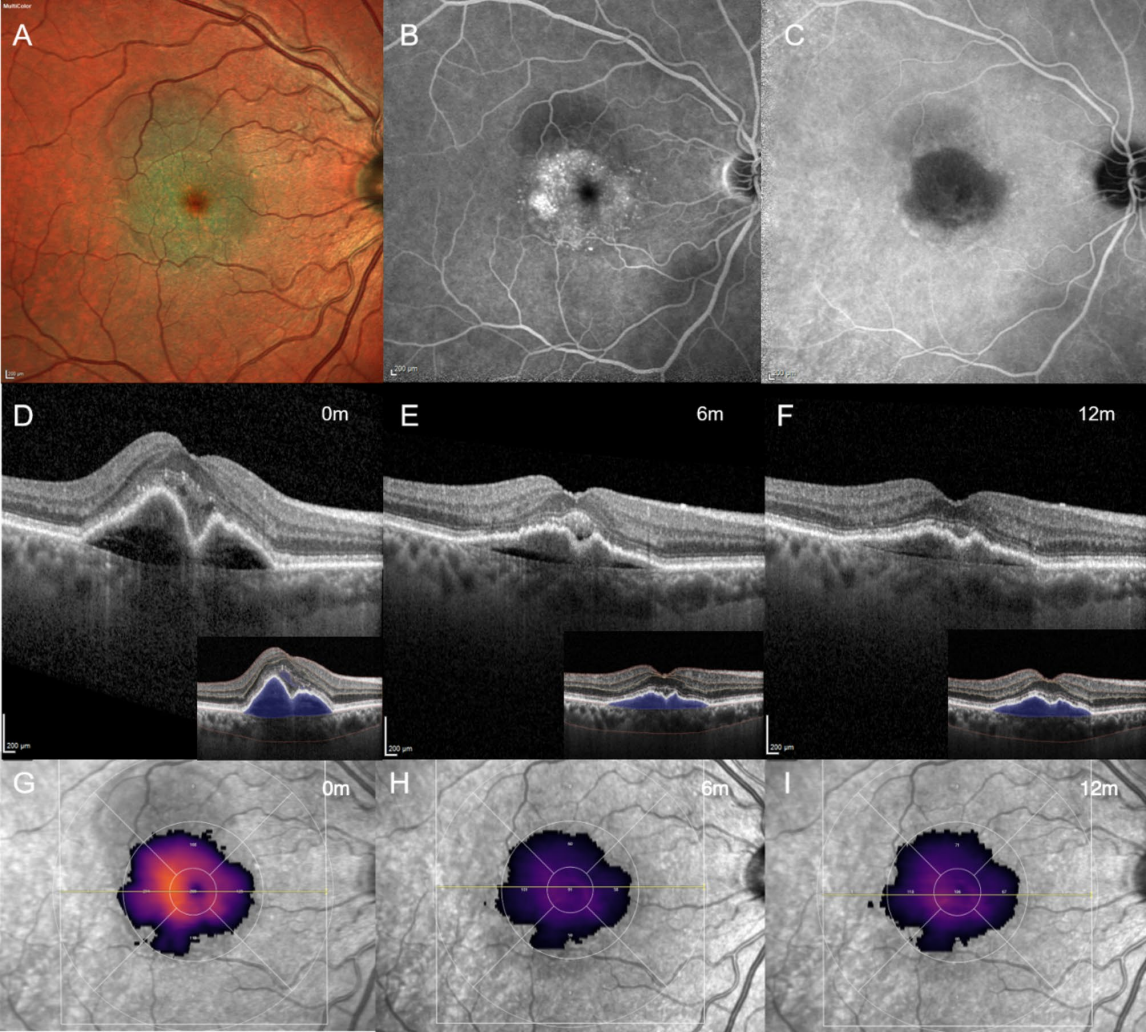
Representation of the longitudinal changes in pigment epithelium detachment (PED) volume in a treatment-naïve patient. Images obtained from the Discovery^®^ platform (Discovery OCT Fluid and Biomarker Detector, RetinAI AG, Switzerland). (Top row) Baseline multimodal imaging of exudative Type 1 macular neovascularization (T1 MNV). (**A**) Multicolor imaging shows the presence of a subfoveal PED associated with subretinal fluid (SRF); (**B**) fluorescein angiography (FA) and (**C**) and indocyanine green angiography (ICGA) confirm the presence of neovessels in the sub-RPE space; (Middle row) (**D**) baseline horizontal optical coherence tomography (OCT) scan shows the presence of subretinal hyperreflective material (SHRM) overlying a fibrovascular PED (insert: segmentation in blue indicates PED volume); (**E**) 6-month follow-up examination displays a decreasing trend of PED volume, with residual subretinal exudation; (**F**) a stabilization of the T1 MNV complex is observed at final follow-up; (Lower row) (**G-H-I**) en-face SLO infrared (IR) images with superimposed colorimetric scale of PED Volume display the longitudinal changes after IVT Faricimab treatment (total 8 injections, q8w after loading phase)

**Fig. 4:**
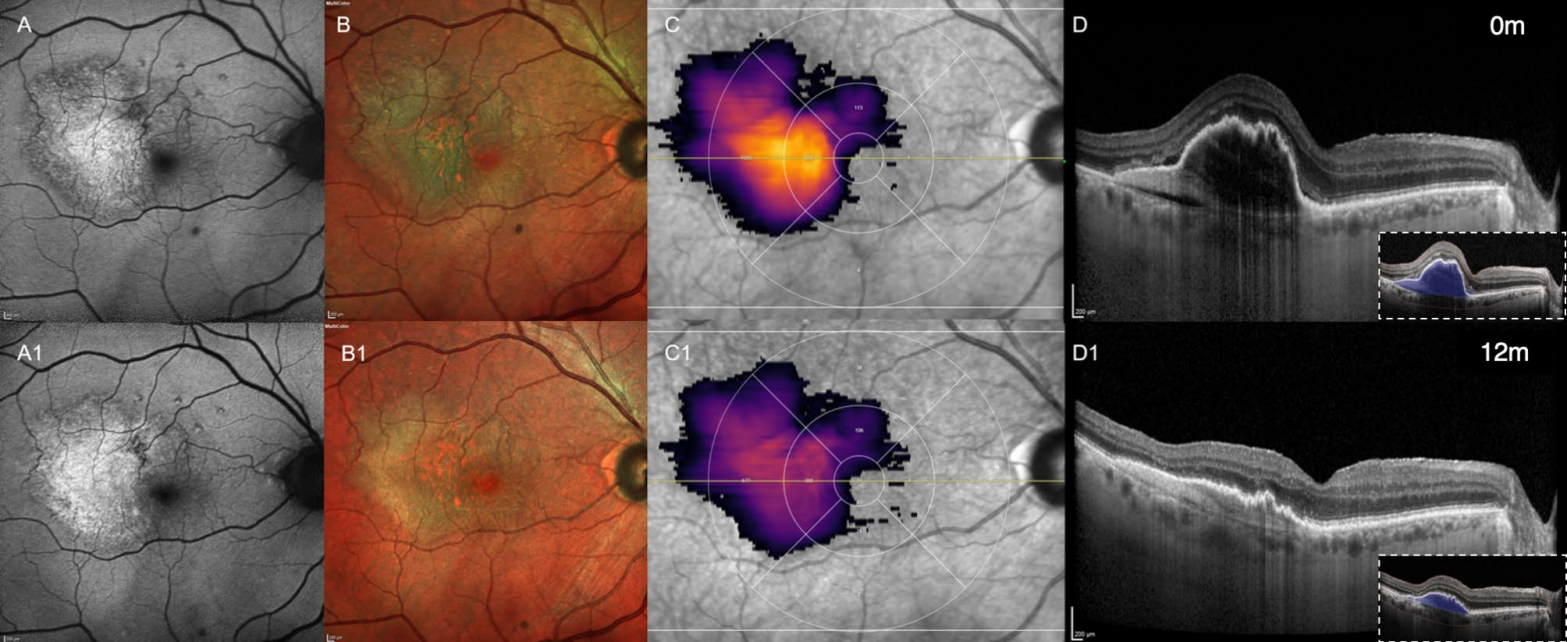
Representation of the longitudinal changes in pigment epithelium detachment (PED) volume in a switch patient (previous 21 anti-VEGF injections). PED’s volume segmentation is represented in blue (inset with dashed lines in D and D1). Images obtained from the Discovery^®^ platform (Discovery OCT Fluid and Biomarker Detector, RetinAI AG, Switzerland). (Top row) Baseline multimodal imaging of exudative Type 1 macular neovascularization (T1 MNV). (**A**) Fundus autofluorescence (FAF) and (**B**) Multicolor imaging shows the presence of hyper-autofluorescent RPE elevation corresponding to a large PED associated with neovessels in the sub-RPE space; (**C**) en-face infrared (IR) with superimposed colorimetric scale of PED Volume and (**D**) horizontal optical coherence tomography (OCT) scan shows the presence of subretinal fluid (SRF) at the edge of a PED and the presence of RPE folds at the apex of the lesion. (Bottom row) (A1-D1) 12-month follow-up shows a noteworthy decrease in the volume of the lesion after switch to intravitreal Faricimab treatment (total 10 injections, q8w after loading phase)

The primary objective was to assess the 12-month efficacy of intravitreal Faricimab on PED volumes, in both treatment-Naïve (Fig. 3) and previously treated non-responder patients (Fig. 4). Our findings align with previous post hoc analysis of pooled data from the TENAYA and LUCERNE trials, which demonstrated that Faricimab was associated with a more rapid and greater reduction in fluids and PEDs compared to Aflibercept during the head-to-head dosing phase of the studies [13]. Overall, this study shows that treatment with Faricimab positively affected retinal fluid control in both Naïve and Switch patients presenting with T1 MNV lesions, and resulted in a significant reduction of AI-assisted quantification of PED Volumes. These findings support the inclusion of Faricimab in the armamentarium of treatments for T1 MNV, particularly in cases complicated by PED.

We observed a rapid decrease in PED volume at the 3-month follow-up in both groups, particularly in Naïve eyes (*p* = 0.049). This may be attributed to the pronounced exudation typically associated with a recently developed lesion, compared to a chronic, previously treated lesion. However, even in the Switch eyes, there was a reduction in PED volume, sustained throughout the follow-up period (Fig. 2). This reduction not only suggests a decrease in fluid accumulation in the sub-RPE compartment, which is isolated and appears to exhibit a limited therapeutic response to anti-VEGF mono-therapy [20], but also indicates a potential anti-fibrotic effect on regression of the fibrovascular component contributing to the PED formation, due to the dual-target inhibition with Faricimab. These findings underscore the multifaceted impact of Faricimab treatment on the structural and functional aspects of MNV pathology, highlighting the need for comprehensive assessment of both fluid and fibrovascular components in evaluating treatment response.

The first real-world study on Faricimab by Stanga et al. [21](11 eyes of 9 patients) demonstrated that SRF, IRF, and PED at baseline were present in 8/11, 3/11 and 8/11 eyes, respectively. After the first Faricimab injection, SRF and IRF were completely resolved in 75% (6/8) and 33% (1/3) of eyes, respectively, and morphological changes such as PED flattening were observed in all affected eyes (8/8). Schneider et al. [22] reported a series of 50 eyes from 46 nAMD patients previously treated for at least 3 times with Aflibercept q4W with persistent fluids that were switched to Faricimab. At 1 month, 84% of eyes showed fluid decrease (responder group), with 33% demonstrating complete fluid resolution and both central retinal thickness (median: −31 μm, *p*-value < 0.01) and PED height (median: −21 μm, *p*-value < 0.01) reduction. A recent paper by Szigiato et al. [15], reported that switching to Faricimab resulted in a reduction in mean central subfield thickness (-11.6 μm, *p*-value = 0.01) and PED height (-44.2 μm, *p*-value = 0.01) after 3 injections, with stable VA and at a similar treatment interval to prior anti-VEGF therapy. Veritti et al. [23] recently published a study demonstrating that Faricimab has an early and substantial efficacy in reducing PED volumes and fluids in treatment-naïve eyes affected by both type 1 and type 3 MNV. The study included 22 eyes (16 with Type 1 MNV and 6 with Type 3 MNV) with a relatively short follow-up period of 120 days and demonstrated a significant and rapid reduction in both mean subretinal fluid (SRF) and intraretinal fluid (IRF). On average, SRF decreased by 14.4% on Day 1, 59.7% on Day 7, and 91.2% on Day 14, while IRF showed a reduction of 23.5% on Day 1, 71.9% on Day 7, and 90.7% on Day 14. These findings suggest that Faricimab may offer substantial and rapid morphological improvements, with enhanced visual acuity outcomes and a favorable safety profile in patients with PEDs associated with nAMD. We agree with the observed trend of early reduction in fluid and PED volumes following Faricimab injections, which, in our experience, is sustained over a period of 12 months.

Our study is not directly comparable to these recent Faricimab studies due to differences in patient inclusion criteria. Unlike these studies, which likely encompassed a broader range of nAMD neovascular lesions, our cohort specifically focused on patients with T1 MNV with PED. This targeted approach allowed us to investigate the efficacy of Faricimab in managing both inward and outward retinal exudation components over 12 months follow-up period, as evidenced by the decrease in IRF and SRF volumes. This reduction was consistent across both groups, indicating the efficacy of Faricimab in controlling disease activity in both Naïve and previously treated Switch eyes.

In our study, BCVA improved at the 12-month follow-up in Naïve eyes (+ 6.9 ETDRS letters from baseline, *p* = 0.053) and remained stable in Switch eyes, despite the Switch group presented a higher baseline BCVA. This difference may be attributed to prior treatments in the Switch group, which had partially managed the exudative component but failed to achieve complete anatomical resolution. In contrast, the naïve group exhibited lower baseline BCVA due to the more active exudative lesions but demonstrated greater improvement throughout the study period with a smaller number of injections.

Indeed, if we evaluate the number of injections performed in the two groups, we found that it was significantly higher in pretreated switch patients (*p* = 0.009). As reported in the literature, in chronically active nAMD, there may be changes in retinal structure and inflammation that alter pharmacokinetics and pharmacodynamics. These include higher concentrations of VEGF, upregulation of other growth factors, vascular endothelial cell mutations, chronic inflammation and even neutralizing antibodies against anti-VEGF [24] leading to a more difficult response to treatment. On the other hand, despite the high number of previous treatments and the chronic and mature stage of the disease, the Switch group showed positive outcomes, supporting the efficacy of Faricimab also in these hard to treat patients.

In our cohort (65 eyes of 65 patients), Faricimab was well tolerated with no instance of IOI episodes recorded during the follow-up period. However, we emphasize the importance of proactive screening to monitor for potential acute IOI episodes during follow-up visits after any type of intravitreal injection, including Faricimab, to ensure overall treatment safety.

AI-driven retinal image analysis is transforming ophthalmology, especially in managing AMD [17, 18]. AI models, particularly those using deep learning (DL) and machine learning (ML), are proving highly effective in measuring biomarkers, forecasting disease progression, and supporting treatment decisions [25]. Recent research has increasingly focused on the quantitative analysis of OCT images using AI algorithms. Schlegl et al. developed a deep learning network capable of automatically quantify retinal fluids [26], , Erfurth et al. used a deep learning algorithm to identify and quantify retinal exudations, and explored the relationship between the fluid volume and visual function following intravitreal injections in AMD patients [27]. Moraes et al. further advanced the field by incorporating additional biomarkers like hyperreflective foci, reinforcing AI’s clinical relevance [28].

Aligned with recent literature, our study underscores the pivotal role of AI in assessing anti-VEGF treatment for AMD through precise measurement of IRF, SRF, and PED volumes. By facilitating accurate quantification of volumetric data, AI provides clinicians with valuable insights into disease management that surpass those offered by traditional methods [29].

This study has several limitations that warrant discussion. Its retrospective design may introduce inherent biases related to data collection and analysis. The relatively small patient cohort limits the statistical power and generalizability of the findings. Additionally, the Switch group had a high mean number of prior injections, which may have influenced treatment outcomes. Furthermore, while the study focuses on PED volume reduction in Type 1 MNV, it does not explore the potential correlation between specific anatomic features, such as intraretinal fluid changes, and vision recovery—a topic that could be addressed in future investigations.

## Conclusions

In conclusion, the study provides compelling evidence for the efficacy of intravitreal Faricimab in reducing PED volumes in T1 MNV secondary to nAMD, reaffirming its role as a valuable therapeutic option in the management of this sight-threatening condition. Moreover, the integration of AI-assisted retinal analysis underscores the transformative potential of technology in enhancing our understanding of retinal diseases, and ultimately our patient care and clinical outcomes. Looking forward, the continued evolution of AI-driven diagnostic tools and advanced imaging modalities offers significant promise for improving diagnostic accuracy and refining treatment approaches in retinal disease management.

## Data Availability

No datasets were generated or analysed during the current study.

## Abbreviations

AI: Artificial intelligence
Anti-VEGF: Anti-vascular endothelial growth factor agents
BCVA: Best-corrected visual acuity
BM: Bruch’s membrane
CC: Choriocapillaris
cRORA: Complete RPE and Outer Retinal Atrophy
CRT: Central retinal thickness
iAMD: Intermediate age-related macular degeneration
IRF: Intraretinal fluid
IVT: Intravitreal injections
nAMD: Neovascular age-related macular degeneration
PED: Pigment epithelium detachment
RPE: Retinal pigment epithelium
SHRM: Subretinal hyper-reflective material
SRF: Subretinal fluid
T1 MNV: Type 1 Macular Neovascularization
